# Exploring healthcare professionals’ views on integrative Chinese–Western medicine in the nutritional management of cancer patients: a qualitative study

**DOI:** 10.3389/fnut.2026.1623146

**Published:** 2026-06-10

**Authors:** Shihua Liu, Guijiao Lin, Xiangyu Peng, Xinlei Wu, Weina Wang, Jiayi Lin, Yanjuan Lin, Liu Yang

**Affiliations:** 1 School of Nursing, Fujian University of Traditional Chinese Medicine, Fuzhou, Fujian, China; 2 Department of Vascular Surgery, Fujian Provincial People’s Hospital, Fuzhou, Fujian, China; 3 Nursing Department, Fujian Medical University Union Hospital, Fuzhou, Fujian, China

**Keywords:** integrative Chinese–Western medicine, cancer, nutritional management, healthcare professionals, qualitative research

## Abstract

**Background:**

Malnutrition is highly prevalent among cancer patients and significantly impairs recovery and treatment outcomes. In China, Integrative Chinese–Western Nutritional Management (ICWNM) has gained increasing attention from healthcare professionals (HCPs) and has been progressively applied in oncology care. Nevertheless, the experiences of HCPs involved in implementing this integrative approach remain largely unexplored.

**Objective:**

To explore the experiences, challenges, and coping strategies of HCPs involved in ICWNM, with the goal of generating insights to strengthen clinical practice and inform policy development.

**Design:**

A qualitative study employing a phenomenological approach was conducted.

**Participants:**

Sixteen HCPs—including six physicians, seven nurses, and three dietitians—with practical experience in oncology nutrition management were purposively recruited until data saturation was achieved.

**Methods:**

Data were collected via semi-structured interviews, which were audio-recorded, transcribed verbatim, and anonymized. The data were analyzed using Colaizz’s seven-step phenomenological approach, followed by inductive thematic analysis to identify and refine themes and subthemes. Analytical rigor was ensured through independent dual coding and member checking.

**Results:**

Four overarching themes and eight subthemes were identified: (1) Professional Fulfillment Coexisting with Stress, with subthemes of *Professional Identity and Self-Efficacy* and *Workload and Cognitive Pressure*; (2) Dual Challenges of Theoretical and Practical Competence, with subthemes of *Limitations of Individual Learning and Peer Sharing* and *Urgent Need for Systematic Professional Training*; (3) Patient Adherence as an Important Link in Nutritional Management, with subthemes of *Multiple Factors Influencing Adherence* and *The Impact of Adherence on Clinical Outcomes*; and (4) Expectations for Clinical Application of ICWNM, with subthemes of *Building a Clinical System Integrating Standardization and Individualization* and *Promoting Sustainable Development through Technological, Policy, and Economic Synergy*.

**Conclusion:**

HCPs experience a sustained cognitive–emotional tension that is shaped by the coexistence of professional fulfillment and practical constraints. The study proposes an integrated analytical framework linking HCPs (cognition–emotion), patients (adherence), and system (support) to elucidate the dynamic interactions between professional agency, patient behavior, and institutional environments. Establishing a comprehensive supportive ecosystem integrating standardized policy frameworks, technology-enabled tools, and sustainable economic incentives is crucial for enhancing the quality, feasibility, and sustainability of ICWNM in clinical practice.

## 1 Introduction

Cancer remains one of the leading causes of morbidity and mortality worldwide, posing a persistent challenge to global health systems. Malnutrition affects approximately 40–80% of oncology patients, profoundly impairing clinical outcomes, reducing treatment tolerance, and diminishing overall quality of life (1–3). In response, international and national medical and nutrition societies have released a series of guidelines and expert consensus statements underscoring the significant role of nutrition in oncology and offering evidence-based recommendations for clinical practice (4–7). Although modern medical nutrition management emphasizes comprehensive assessment and individualized dietary planning based on evidence-based principles to improve patients’ nutritional status (8), in practice it often overlooks the holistic functional properties of food, focusing on isolated nutrients rather than whole-food synergy—and relies excessively on nutritional supplements (9, 10). Within China’s long-standing tradition of Traditional Chinese Medicine (TCM) dietary culture, these limitations have prompted growing interest in Integrative Chinese–Western Nutritional Management (ICWNM) among healthcare professionals (HCPs) seeking more appropriate and culturally aligned cancer nutrition strategies.

ICWNM represents a comprehensive care model that integrates modern nutritional science with TCM dietary therapy, guided by the principles of “treating disease before its onset” (zhi wei bing) and “syndrome-based diet formulation” (bianzheng shishan). Its core philosophy harmonizes individualization and holism, formulating diets based on syndrome differentiation and adjusting nourishment according to body constitution, thereby unifying targeted interventions with overall nutritional support.

Traditional Chinese Medicine (TCM) dietary therapy, with a theoretical foundation spanning thousands of years, provides a complementary and cross-paradigm medical practice grounded in syndrome differentiation and holistic regulation. It emphasizes individualized, dynamically adjusted, and seasonally adapted nutritional interventions aligned with disease progression (11). This approach has attracted increasing attention in chronic disease rehabilitation and demonstrates unique advantages in addressing cancer-related malnutrition. Consequently, integrative nutritional programs combining TCM and Western nutritional science have been widely implemented in comprehensive hospitals both in China and internationally, aiming to enhance clinical efficacy and improve quality of life (12, 13). In China, national health policies have continued to strengthen the strategic role of TCM, establishing the integration of Chinese and Western medicine as a fundamental component of the national healthcare system (14). Globally, the World Health Organization (WHO) has advocated for the evidence-based and standardized application of traditional medicine, providing an international framework for advancing integrative nutrition management (15). Together, these initiatives underscore both the significant potential and urgent necessity of promoting synergistic Chinese–Western nutritional practices in modern oncology care.

**Figure 1:**
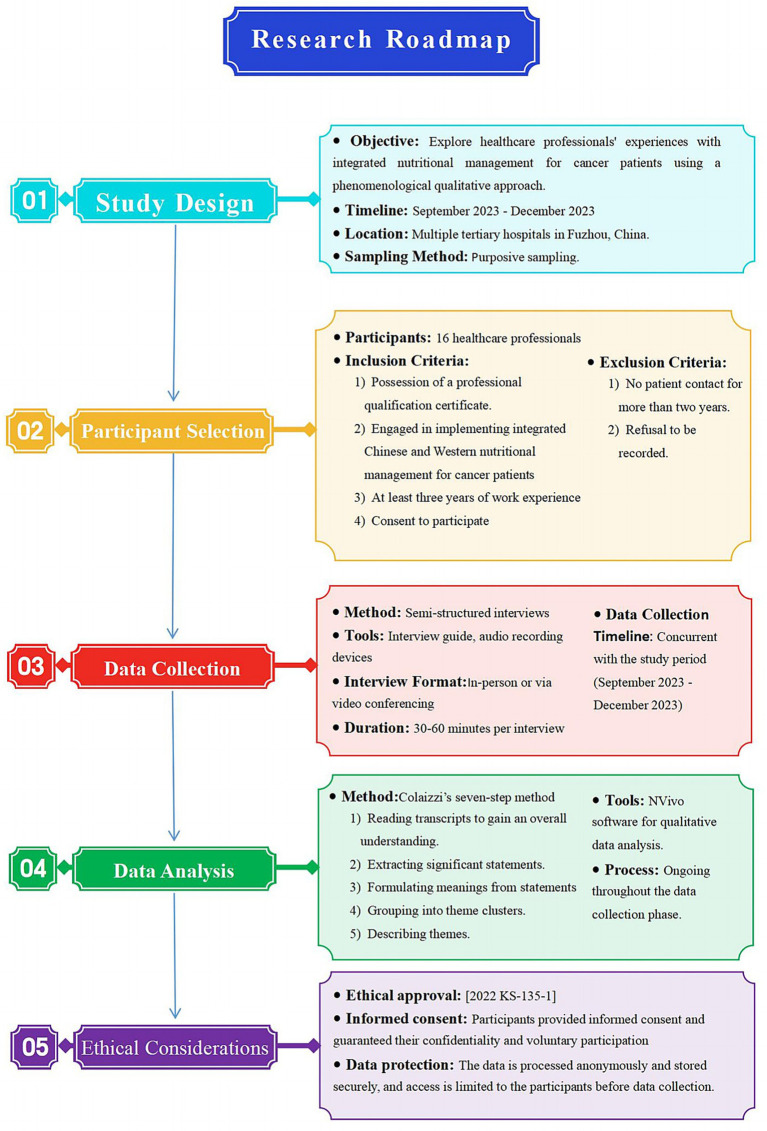
Technical roadmap of the study on healthcare professionals’ experiences in ICWNM. The figure illustrates the overall workflow of the study, covering five sequential stages: (1) Research design, (2) participant recruitment, (3) data collection, (4) data analysis, and (5) ethical considerations. ICWNM, integrative Chinese–Western nutritional management; HCPs, healthcare professionals; TCM, traditional Chinese medicine.

Despite growing clinical demand and policy momentum, the implementation of ICWNM still faces multiple challenges. There is a lack of systematic qualitative evidence capturing the practical barriers, enabling factors, and emotional experiences of frontline HCPs engaged in this integrative process (16). Understanding these lived experiences is crucial for identifying actionable strategies, clarifying determinants of successful integration, and optimizing interdisciplinary collaboration pathways in integrative oncology nutrition. Therefore, this study adopted a phenomenological qualitative design (17), using semi-structured interviews to explore HCPs’ experiences in implementing ICWNM for cancer patients. The study aimed to identify the core challenges, adaptive strategies, and expectations perceived by practitioners, thereby providing empirical insights to inform clinical optimization and policy development for enhancing both therapeutic effectiveness and patient well-being (see Figure 1).

## 2 Methods

This study adopted a phenomenological qualitative design and was reported in accordance with the Consolidated Criteria for Reporting Qualitative Research (COREQ) guidelines (Appendix 1 in Supplementary material) (18). Ethical approval was obtained from the Ethics Committee of the Third Affiliated People’s Hospital of Fujian University of Traditional Chinese Medicine (Approval No. 2022-k1-013).

### 2.1 Participants and recruitment

#### 2.1.1 Participants

A purposive sampling strategy was employed to recruit healthcare professionals (HCPs) who were directly involved in the practice of integrative Chinese–Western nutritional management (ICWNM) for cancer patients, including physicians, nurses, and dietitians.

Inclusion criteria were as follows: (1) clinical physicians, registered nurses, or registered dietitians holding valid professional licenses and corresponding professional titles; (2) having engaged continuously for at least 3 years in oncology nutrition management or cancer care, and regularly participating in or leading ICWNM interventions (e.g., TCM dietary therapy, pattern-based diet formulation, syndrome-based nutritional adjustment, or guidance on integrative nutrition assessment and support planning); (3) willingness to participate in audio-recorded interviews and to sign written informed consent.

Exclusion criteria included: HCPs not currently involved in direct clinical care (e.g., on extended leave or primarily engaged in administrative duties).

#### 2.1.2 Recruitment procedures

Recruitment was conducted by a female researcher trained in qualitative methodology with a background in nursing and integrative medicine. Recruitment proceeded concurrently through both channels without additional quantitative sampling requirements. First, through organizational coordination, the researcher contacted target hospital departments and distributed study information via email, internal communication platforms, social media, and Tencent Meeting. HCPs interested in participation could contact the research team directly. Second, through network referral, potential participants were recommended via the researchers’ academic and institutional networks. The recruitment process began in September 2023 and continued until data saturation was achieved—defined as the point at which no new experience types or analytical themes emerged in consecutive interviews (19). By December 2023, a total of 16 HCPs were recruited, and all interview data had been collected.

### 2.2 Interview implementation

#### 2.2.1 Development of the interview guide

The semi-structured interview guide was developed around the practical context of ICWNM and informed by literature on integrative medicine, health behavior, and nursing education. The guide was refined through a three-round iterative process to ensure scientific rigor and logical coherence. Round 1: Based on a systematic literature review and research objectives, the research team drafted a preliminary framework aligning key topics with study questions. Round 2: Three experts with extensive ICWNM experience—including a clinical physician, a nursing education specialist, and a nutrition expert—assessed the content validity of the guide. The content validity index (CVI) was 0.92, and feedback was used to revise the structure and wording of items. Round 3: Three HCPs from different professional backgrounds participated in pilot interviews to test logical flow, semantic clarity, and feasibility. The guide was further revised based on pilot feedback, resulting in the final version.

The finalized guide comprised five thematic areas: (1) Professional experiences and emotional perceptions—HCPs’ understanding of their roles and psychological experiences in ICWNM; (2) Evolution of practice—how integrative nutrition practices have developed over time; (3) Implementation challenges—barriers encountered and coping strategies; (4) Professional training and peer sharing—needs and suggestions regarding training content, formats, and interdisciplinary exchange; (5) Policy support and future expectations—perspectives on policy frameworks, digital technology, and system development. Each topic included two to three open-ended questions and corresponding probes to elicit detailed responses. The complete guide is provided in Appendix 2 in Supplementary material.

#### 2.2.2 Interview procedures and settings

All interviews were conducted by two researchers trained in qualitative methods, both with nursing and ICWNM backgrounds. Interviews were held either face-to-face or via secure online meetings (Tencent Meeting platform), scheduled at times convenient for participants and avoiding clinical peak hours to ensure a quiet, private, and comfortable setting. Each interview lasted 30–60 min and was audio-recorded with written informed consent.

Interviewers used the semi-structured guide to ask open-ended questions and flexibly adjusted the order and depth of questioning according to participants’ responses. When participants described specific experiences, probing questions (e.g., “Could you tell me more about that situation?” or “How common is this in your practice?”) were used to elicit richer descriptions. Researchers closely observed nonverbal cues—tone, facial expression, pauses, and posture—and annotated them in field notes to aid interpretation. In moments of hesitation or silence, interviewers applied active listening and neutral feedback (e.g., nodding, maintaining eye contact, or brief acknowledgments such as “I understand” or “That’s important”) to encourage continued reflection. At the end of each interview, the researcher summarized key points and invited participants to confirm accuracy or clarify ambiguous statements. All recordings were transcribed verbatim and anonymized using codes (e.g., P1, P2). Two team members independently cross-checked transcripts against recordings to ensure completeness and accuracy.

#### 2.2.3 Ethical considerations and communication principles

All interviews adhered to ethical research principles. Participants were informed of the study’s purpose, confidentiality measures, and voluntary participation rights before interviews, and written informed consent was obtained. They were told they could refuse to answer or withdraw at any time. The research team standardized its communication strategies, using neutral terms such as “nutritional intervention” or “dietary guidance” instead of potentially biased expressions like “dietary restriction” or “treatment failure.” During data collection, interviewers maintained an empathetic and non-judgmental stance, attentively observing emotional cues and ensuring participants felt respected and comfortable. For online sessions, encrypted platforms were used, and all recordings and transcripts were stored on password-protected hard drives accessible only to authorized team members, ensuring data security and confidentiality.

### 2.3 Data processing and quality control

#### 2.3.1 Data processing

All audio recordings were transcribed verbatim by the principal researcher, who had no direct clinical or hierarchical relationship with participants. The transcripts were cross-checked by team members against original recordings to ensure accuracy and completeness. Field notes documenting contextual observations were incorporated into the final transcripts.

Each audio file was assigned a numeric code, and only anonymized transcripts were used for analysis. When unclear or incomplete information was detected, follow-up interviews were conducted with the corresponding participants to verify and deepen understanding of their perspectives.

#### 2.3.2 Quality assurance

To ensure credibility and trustworthiness, three quality-control strategies were implemented: (1) Building rapport—researchers established trust by learning about participants’ professional backgrounds beforehand to minimize defensiveness during interviews; (2) Confidentiality agreements—all participants signed data confidentiality pledges; and (3) Member checking—transcripts or key summaries were returned to selected participants (especially senior HCPs with > 10 years’ experience) for verification before inclusion in analysis.

### 2.4 Data analysis

Data were analyzed using Colaizzi’s seven-step phenomenological analysis method (20).

After verbatim transcription, two researchers independently reviewed the recordings and transcripts to ensure consistency. The research team repeatedly read the texts to extract significant statements relevant to the study objectives, formulated meaning units, and grouped similar units into themes and subthemes through inductive categorization. Following Colaizzi’s phenomenological method, the analytical process further involved developing an exhaustive description of the phenomenon and identifying its fundamental structure. The preliminary thematic interpretation was subsequently refined to ensure conceptual coherence.

Initial coding was performed separately by two researchers, followed by team discussions to resolve discrepancies and reach consensus on a unified thematic framework. Data organization and coding were supported using NVivo 12 software, facilitating systematic management of meaning units and thematic clustering. To verify coding reliability, dual independent coding was applied to approximately 30% of transcripts, yielding an inter-coder reliability coefficient of Cohen’s *κ* = 0.82, indicating high coding consistency.

## 3 Results

### 3.1 Characteristics of participants

A total of 16 healthcare professionals (HCPs) participated in this study, all from five tertiary hospitals in Fuzhou, Fujian Province, including the Fujian Cancer Hospital, Fujian Provincial Hospital (affiliated with Fuzhou University), Second and Third Affiliated Hospitals of Fujian University of Traditional Chinese Medicine, and the Fujian Apitherapy Hospital. Participants were engaged in oncology-related diagnosis, nursing, or nutritional care under the integrative Chinese–Western nutritional management (ICWNM) model. Among them, seven were male and nine female, with an age range of 26–60 years; participants aged below 30 years (37.5%) and 41–50 years (31.3%) accounted for the majority. The sample included six physicians (37.5%), seven nurses (43.8%), and three dietitians (18.8%). Clinical experience ranged from 3 to over 30 years, with five (31.3%) holding junior titles, five (31.3%) intermediate titles, and six (37.5%) senior titles. All HCPs participated in semi-structured interviews, conducted either face-to-face or via Tencent Meeting. The total recording time was approximately 565 min, with each interview lasting 30–60 min (average ≈35 min). After transcription and data cleaning, approximately 120,000 words of textual data were generated for analysis.

Demographic and professional characteristics of participants are summarized in Table 1, and detailed profiles are provided in Supplementary Material 3.

**Table 1:** Demographic characteristics of participants (*N* = 16).

Variable	Category	*n* (%)
Gender	Female	9 (56.25)
	Male	7 (43.75)
Age (years)	26–30	6 (37.50)
	31–40	3 (18.75)
	41–50	5 (31.25)
	56–60	2 (12.50)
Years of work experience	3–5 years	6 (37.50)
	6–15 years	3 (18.75)
	16–30 years	5 (31.25)
	>30 years	2 (12.50)
Affiliated hospitals (all participants)	Fujian Cancer Hospital	3 (18.75)
	Fujian Provincial Hospital, Affiliated to Fuzhou University	3 (18.75)
	The Third Affiliated Hospital of Fujian University of Traditional Chinese Medicine	4 (25.00)
	The Second Affiliated Hospital of Fujian University of Traditional Chinese Medicine	1 (6.25)
	Fujian Apitherapy Hospital	5 (31.25)
Professional category	Physicians (including integrative medicine, internal medicine, surgery, and general medicine)	6 (37.50)
	Nurses	7 (43.75)
	Dietitians	3 (18.75)
Education level	Doctoral degree	1 (6.25)
	Master’s degree	8 (50.00)
	Bachelor’s degree	5 (31.25)
	Associate degree	2 (12.50)
Professional title	Junior	5 (31.25)
	Intermediate	5 (31.25)
	Senior	6 (37.50)

### 3.2 Overview of themes

Through inductive thematic analysis, four major themes and eight subthemes were identified from the interview data (Table 2). Participants’ quotations were selected to illustrate the key findings and ensure representational richness.

**Table 2:** Themes and subthemes reflecting HCPs’ experiences with ICWNM.

Main themes	Subthemes
Theme 1: Professional fulfillment coexisting with stress	Professional identity and self-efficacy
	Workload and cognitive pressure
Theme 2: Dual challenges of theoretical and practical competence	Limitations of individual learning and peer sharing
	Urgent need for systematic professional training
Theme 3: Patient adherence as an important link in nutritional management	Multiple factors influencing adherence
	The impact of adherence on clinical outcomes
Theme 4: Expectations for clinical application of ICWNM	Building a clinical system integrating standardization and individualization
	Promoting sustainable development through technological, policy, and economic synergy

### 3.3 Theme 1: professional fulfillment coexisting with stress

HCPs described their experiences in ICWNM as a dynamic state where professional satisfaction and emotional stress coexist. While many participants reported a sense of achievement and meaning from observing patient recovery and contributing to innovative integrative practices, they simultaneously experienced intense cognitive and emotional strain due to workload, interdisciplinary communication barriers, and the demands of maintaining dual knowledge systems.

#### 3.3.1 Subtheme 1.1: professional identity and self-efficacy

HCPs derived strong professional identity and self-efficacy from patient improvement, recognition from colleagues, and alignment with personal values. As one participant noted, “When patients recover and express gratitude, I feel that all the extra effort was worthwhile” (P15). Another reflected, “Integrative nutrition allows me to use both TCM and modern methods to help patients—it makes me feel more competent and fulfilled” (P11).

#### 3.3.2 Subtheme 1.2: workload and cognitive pressure

Despite such fulfillment, participants reported considerable cognitive load arising from the need to master both biomedical and TCM-based nutritional frameworks. One physician stated, “Sometimes it’s hard to connect the TCM pattern differentiation with modern nutritional assessment—it feels like learning two completely different systems” (P4). Another nurse explained, “We have to coordinate with doctors, dietitians, and patients, which takes extra communication and mental energy” (P1). Several participants highlighted a persistent knowing–doing gap, observing that “we know what to do, but limited time and institutional constraints make it difficult to apply it in practice” (P5, P6).

### 3.4 Theme 2: dual challenges of theoretical and practical competence

HCPs identified significant challenges in transforming conceptual understanding into practical competence. Many felt underprepared to systematically integrate TCM and Western nutritional principles in clinical practice, citing the absence of structured training opportunities and coherent institutional support.

#### 3.4.1 Subtheme 2.1: limitations of individual learning and peer sharing

Participants described current learning patterns as fragmented and self-initiated, often relying on personal experience, informal discussions, or sporadic workshops. As a nurse remarked, “We mainly learn by trial and error or from colleagues’ experience—there’s no standardized platform for learning ICWNM” (P2). This inconsistency contributed to varied clinical practices and hindered effective knowledge translation (P5).

#### 3.4.2 Subtheme 2.2: urgent need for systematic professional training

HCPs unanimously expressed a strong demand for systematic, interdisciplinary, and evidence-based training programs. They emphasized the need for structured curricula, integration of case-based simulations, and cross-disciplinary collaboration. As one physician shared, “We need organized team training that brings together TCM doctors, dietitians, and nurses so that everyone speaks the same language” (P10).

Another participant added, “Formal certification or continuing education would give us confidence and legitimacy when applying ICWNM in practice” (P8).

### 3.5 Theme 3: patient adherence as an important link in nutritional management

HCPs agreed that patient adherence plays a crucial role in determining the success of ICWNM interventions. They observed that adherence levels are shaped by complex, interrelated factors and that nonadherence often compromises the effectiveness of otherwise well-designed plans.

#### 3.5.1 Subtheme 3.1: multiple factors influencing adherence

Participants reported that adherence is influenced by patients’ cognitive understanding, cultural dietary beliefs, financial capacity, and the quality of communication between patients and providers. As one dietitian noted, “Some patients believe more in medications than in food therapy, so they do not follow the plan strictly” (P7). Another added, “Economic stress also affects whether they can afford the recommended ingredients or supplements” (P12). HCPs emphasized that effective communication and empathy are essential for fostering patient motivation and trust (P16, P2).

#### 3.5.2 Subtheme 3.2: the impact of adherence on clinical outcomes

All participants highlighted that patient adherence directly determines the clinical outcomes of nutritional interventions. When adherence is high, patients experience improved nutritional status, better symptom control, and enhanced quality of life. Conversely, poor adherence leads to diminished effects.

Several HCPs also pointed out the lack of standardized adherence monitoring tools, which limits the ability to evaluate and adjust interventions in a timely manner (P8, P11).

### 3.6 Theme 4: expectations for clinical application of ICWNM

Participants expressed forward-looking expectations for the institutionalization and systematization of ICWNM practices, envisioning an integrative framework that aligns standardization with individualization and leverages technology and policy support.

#### 3.6.1 Subtheme 4.1: building a clinical system integrating standardization and individualization

HCPs advocated for a standardized yet flexible clinical system that combines evidence-based guidelines with individualized treatment adjustments. As one physician explained, “We hope for a unified assessment and intervention framework so that each patient receives consistent yet personalized care” (P10). They emphasized that such a system would enhance communication across disciplines and improve continuity of care (P3, P7, P11).

#### 3.6.2 Subtheme 4.2: promoting sustainable development through technological, policy, and economic synergy

Participants underscored that sustainable development of ICWNM requires synergistic support across technological, policy, and economic dimensions.

They highlighted the potential of artificial intelligence (AI) and clinical decision support systems (CDSS) to enhance precision and efficiency, alongside the necessity of policy incentives (e.g., insurance reimbursement) and dedicated financial resources to sustain program implementation (P5, P10, P14).

### 3.7 Conceptual framework

**Figure 2:**
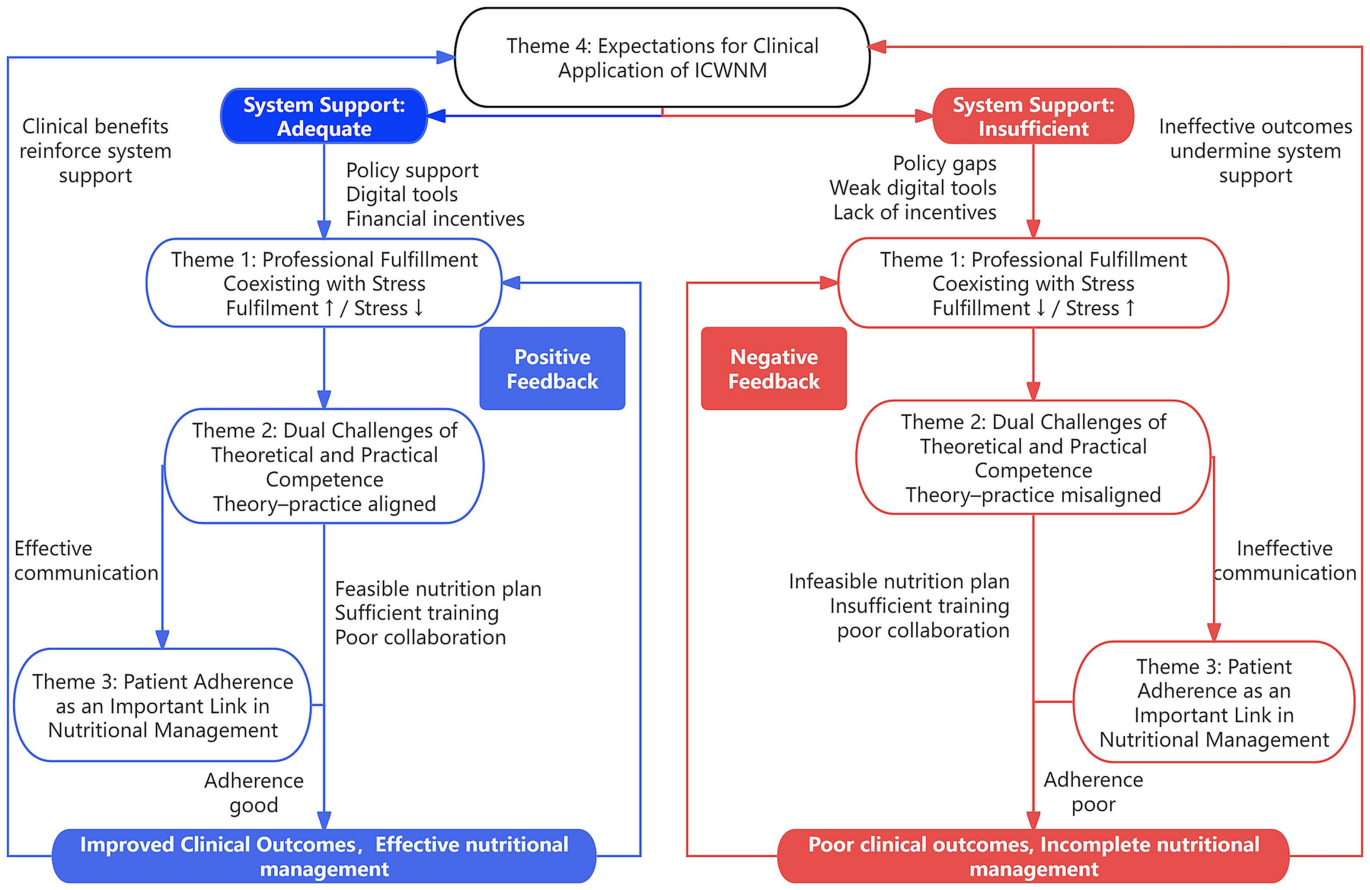
Conceptual framework illustrating the dual-loop relationship among the four themes in Integrative Chinese–Western Nutritional Management (ICWNM). The left (blue) loop represents a positive feedback process under adequate system support, whereas the right (red) loop represents a negative feedback process under insufficient support.

Based on the integrated analysis, a conceptual framework was constructed to visualize the interrelationships among the four major themes identified in this study (Figure 2). The framework depicts two interlinked feedback pathways that illustrate how varying levels of system support influence healthcare professionals (HCPs), patient adherence, and the overall quality of integrative nutritional management.

When system support—such as policy guidance, technological tools, and financial resources—is adequate, Theme 1 (Professional Fulfillment Coexisting with Stress) tends to evolve toward positive engagement, reinforced by Theme 2 (Dual Challenges of Theoretical and Practical Competence), Theme 3 (Patient Adherence as an Important Link in Nutritional Management), and Theme 4 (Expectations for Clinical Application of ICWNM). This interaction fosters effective communication, feasible nutrition planning, and improved clinical outcomes, forming a positive feedback loop. In contrast, when systemic support is insufficient, professional pressure intensifies, theory–practice alignment weakens, and patient adherence declines, giving rise to a negative feedback loop characterized by limited competence, reduced motivation, and suboptimal nutritional management. This dual-loop framework captures the dynamic interplay between professional effort, patient participation, and system-level resources as described by participants from different institutions.

## 4 Discussion

Through in-depth interviews with 16 healthcare professionals (HCPs) from five tertiary hospitals, this study provides an interpretive synthesis of the multifaceted experiences of practitioners engaged in integrative Chinese–Western nutritional management (ICWNM).

The findings reveal a pervasive cognitive–emotional tension among HCPs, characterized by the coexistence of professional fulfillment and work-related stress. On one hand, participants derived a strong sense of satisfaction and self-efficacy from witnessing patient recovery and improved outcomes (21, 22). On the other hand, they experienced persistent cognitive load and emotional strain stemming from the absence of structured training systems, the complexity of dual knowledge frameworks, and obstacles to interdisciplinary collaboration.

This duality reflects an adaptive psychological process in which HCPs seek to reconcile their recognition of the value of integrative care with the challenges of its implementation—a dynamic mechanism that drives ongoing learning, professional development, and institutional adaptation.

Furthermore, patient adherence emerged as a pivotal link between professional effort and clinical effectiveness, whereas deficiencies in policy, technology, and economic support functioned as structural bottlenecks limiting ICWNM’s broader adoption.

Synthesizing these findings, the study proposes an integrated analytical framework encompassing HCP cognition–emotion, patient adherence, and system support, capturing the dynamic interplay among professional agency, patient behavior, and institutional context.

This framework not only deepens understanding of ICWNM’s complexity but also provides theoretical and practical guidance for building feasible and sustainable implementation pathways.

### 4.1 Cognitive–emotional tension: coexistence of professional fulfillment and pressure

HCPs in this study demonstrated a complex psychological state characterized by the simultaneous presence of professional fulfillment and work-related stress. While the sense of fulfillment nurtured positive emotions and work engagement, stress commonly arose from insufficient mastery of knowledge, role ambiguity, and limited time resources. Together, these dual forces formed a persistent cognitive–emotional tension—not merely an inner conflict, but a dynamic balancing process through which HCPs rationally affirmed the value of integrative care while managing practical constraints. Such tension represents a common psychological pattern among professionals working within integrative healthcare environments (23).

Positive clinical outcomes—such as patients’ improved nutritional status, vitality, and recovery—elicited a strong sense of professional accomplishment and reinforced practitioners’ identity, consistent with Bandura’s self-efficacy theory (24). Positive feedback from patients further strengthened HCPs’ sense of professional value, serving as a key psychological resource that motivated continuous learning and engagement. Similarly, prior studies have shown that an enhanced professional identity reinforces team cohesion and motivation, thereby promoting the stability of integrative healthcare models (22).

However, this rewarding experience was frequently offset by heavy workload and sustained cognitive strain. Many participants described the challenge of mastering two parallel theoretical paradigms—Traditional Chinese Medicine (TCM) and Western nutrition science—which increased the complexity of knowledge integration and clinical decision-making. The demand for interdisciplinary collaboration and individualized interventions further intensified this “knowing–doing gap,” exposing the discrepancy between theoretical understanding and clinical feasibility (25). These observations align with the Job Demands–Resources (JD–R) model, which posits that when job demands escalate in the absence of adequate organizational resources, individuals are more susceptible to emotional exhaustion and professional burnout (26).

This tension was further exacerbated by system-level factors. Although interdisciplinary collaboration facilitates knowledge exchange, discrepancies in professional values and communication styles often resulted in decision delays or conceptual disagreements. Empirical evidence suggests that psychological strain in integrative practice commonly arises from role ambiguity and value incongruence, particularly when unified institutional frameworks are lacking (27). Participants noted that the absence of structured training and formal collaboration platforms fostered fragmented and isolated learning, compounding uncertainty and stress. Importantly, this coexistence of achievement and pressure is not inherently detrimental; a moderate level of cognitive–emotional tension may function as a constructive driving force, stimulating competence development and clinical innovation (28). Nevertheless, without adequate organizational and psychological support, this positive tension can easily shift toward exhaustion, undermining both sustainability and team cohesion.

Therefore, future strategies should address organizational support and mental health management by establishing structured training systems, providing accessible counseling channels, and cultivating a collaborative culture that reinforces professional resilience and long-term engagement.

### 4.2 Professional training and capacity building

HCPs demonstrated strong motivation for continuous learning and knowledge integration in ICWNM. However, due to limited institutional resources, their learning processes remained largely self-initiated, fragmented, and individualized. As a result, clinical competence often depended more on personal initiative than on structured institutional training. Over time, this self-directed model risks hindering the sustainable development of professional capacity.

Meta-analytic evidence indicates that in interdisciplinary healthcare settings, professional capacity building frequently relies on self-study, peer consultation, and experiential learning. Yet, the absence of standardized curricula, training frameworks, and evaluation mechanisms constrains the consistency and transferability of knowledge (29). Although such individually driven learning can temporarily bridge knowledge gaps, it lacks continuity and systematic reinforcement, thereby limiting the long-term quality and scalability of ICWNM practice.

According to Boud and Hager’s theory of Continuing Professional Development (CPD), professional growth is a dynamic and cyclical process requiring the interplay of individual learning, organizational support, and institutional assurance (30). Compared with mature international training systems, China currently lacks well-structured educational programs dedicated to ICWNM, resulting in delays in knowledge dissemination, competency assessment, and clinical translation (31, 32). Previous studies have shown that standardized curricula, simulation-based instruction, and interdisciplinary workshops significantly enhance teamwork, implementation fidelity, and the institutionalization of knowledge (33, 34). Therefore, a system-driven training framework should replace fragmented self-learning, fostering a transition from individual to team-based capacity building.

To this end, universities and integrative hospitals should jointly develop cross-disciplinary training programs that integrate TCM dietary therapy, Western nutritional assessment, and oncologic metabolism management into a unified curriculum. These programs should be supplemented with case-based learning, simulation exercises, and supervised clinical practicums to bridge theory and practice.

Furthermore, hospitals should allocate protected training time within staff schedules and introduce policy-linked incentives—such as continuing education credits—to ensure participation, consistency, and institutional continuity (35). Collectively, these measures would strengthen both the practical relevance and sustainability of professional capacity-building efforts, laying the foundation for standardized and scalable ICWNM implementation.

### 4.3 Patient adherence as the bridge between intervention and outcomes

This study highlights patient adherence as a central mediating factor linking the design of nutritional interventions to their ultimate clinical effectiveness. HCPs consistently emphasized that while nutritional planning marks the starting point of ICWNM, the true endpoint lies in patients’ sustained and accurate implementation of prescribed guidance. Therefore, the clinical value of ICWNM depends not only on the scientific design of nutrition plans but also on the extent to which patients transform recommendations into consistent, self-directed behaviors.

Participants identified multiple determinants of adherence, including personal beliefs, cultural habits, economic constraints, and social support systems. These observations align with prior evidence suggesting that patient behavior is co-shaped by individual motivation and sociocultural context (36). From a psychological perspective, trust and understanding of therapeutic approaches emerged as pivotal factors influencing adherence. Patients who lacked knowledge of TCM dietary principles or regarded medication as the sole effective treatment tended to undervalue nutritional interventions. According to the Health Belief Model (HBM) (37), weak perceptions of disease severity, limited recognition of benefits, and high perceived barriers significantly reduce the persistence of health-related behaviors. Several HCPs observed that adherence was more likely to be maintained once patients personally experienced tangible therapeutic benefits. Moreover, cultural factors exerted a profound influence on adherence. TCM dietary therapy emphasizes body constitution differentiation and holistic balance, which may conflict with entrenched dietary preferences or traditional “food-as-medicine” beliefs. Older adults, in particular, often relied on experiential knowledge rather than professional advice, thereby diminishing the efficacy of interventions. Studies conducted in East Asia have similarly shown that when medical recommendations contradict local food culture, patients frequently demonstrate surface compliance or symbolic adherence (38). Consequently, effective ICWNM must incorporate culturally sensitive communication strategies, aligning evidence-based recommendations with patients’ lived experiences, beliefs, and value systems to foster understanding, acceptance, and long-term behavioral engagement.

In addition, adherence barriers extend beyond patient-level factors. When nutrition plans overlook patients’ economic realities, family support, or lifestyle patterns, compliance becomes difficult to sustain. The complexity of food preparation and associated costs often deter patients from maintaining dietary regimens over time (39). Therefore, implementing Shared Decision-Making (SDM) between clinicians and patients is crucial. International evidence demonstrates that SDM enhances patient engagement, strengthens therapeutic alliance, and improves the continuity of healthy behaviors (40). Incorporating SDM into ICWNM provides a practical communication model that empowers patients as active partners in their nutritional care, thereby bridging the gap between clinical intention and behavioral execution.

### 4.4 Systemic barriers: policy, technology, and economics

At a macro level, the persistent challenges facing ICWNM reflect structural deficits in policy, technology, and economic support systems rather than isolated professional or behavioral issues. Global research on integrative medicine indicates that when institutional environments lack stable training systems, operational technologies, and rational financial incentives, integrative models often struggle to transition from pilot programs to routine clinical practice (41).

At the policy level, the absence of institutionalized guidelines remains a fundamental barrier. Participants emphasized substantial variability across institutions in both the quality and standardization of ICWNM, where inadequate training and regulatory frameworks have left practice largely experience-based. Similar patterns were noted in the United Kingdom prior to the introduction of national nutritional-care guidance: in the absence of unified standards and professional certification, screening and implementation were highly variable across institutions, whereas the rollout of NICE guidance and national campaigns subsequently improved the standardization and uptake of nutrition screening and support (42). Standardized clinical pathways thus function not only as technical references but also as mechanisms of professional legitimacy, clarifying interdisciplinary boundaries and responsibilities. HCPs’ demand for policy support reflects their pursuit of institutional legitimacy, as well as protected time and resources to mitigate long-term cognitive and emotional burden.

Technologically, the lack of integrated tools contributes to fragmented knowledge, operational gaps, and the persistent knowing–doing divide. The cognitive pressure and collaboration difficulties described by HCPs partly arise from the absence of a translation mechanism bridging TCM diagnostic logic with Western nutritional assessment. International evidence demonstrates that AI-based Clinical Decision Support Systems (CDSS) can integrate metabolic indicators and patient data, reducing reliance on individual experience and enhancing the consistency of oncologic nutrition care (43). However, China currently lacks digital platforms capable of merging TCM body constitution data with modern nutritional metrics. Consequently, HCPs must manually synthesize multiple information sources, resulting in data fragmentation, time pressure, and delayed adherence monitoring. This situation represents a knowledge translation barrier—HCPs understand how ICWNM should operate in theory but cannot implement it effectively due to the absence of unified workflows and supportive technological infrastructure (44). Bridging this gap requires the creation of both technical and institutional conditions that make knowledge application operational. Future digital innovation should focus on actionable knowledge translation, developing integrated CDSS platforms that combine TCM parameters, nutritional indicators, and behavioral data to enable the bidirectional flow between theory and practice.

Economically, the absence of reimbursement and incentive mechanisms further constrains implementation. Participants consistently reported that the lack of insurance coverage and performance-based rewards made ICWNM difficult to sustain. Empirical evidence suggests that integrating nutritional interventions into insurance payment systems can increase patient adherence by 20–30% (45). In China, however, most ICWNM programs still rely on temporary research grants, lacking stable financial support. This has created an “effort–burden paradox,” wherein additional workload and time investment translate into personal costs, thereby undermining professional motivation. Establishing bidirectional incentive mechanisms is therefore critical: on one hand, gradually expanding insurance coverage for ICWNM; on the other, embedding integrative nutrition indicators into hospital performance evaluations to ensure institutional recognition and sustainable motivation.

In summary, policy, technology, and economics form an interdependent triad supporting the sustainable development of ICWNM. Policy provides top-level legitimacy, technology enables knowledge translation and operational efficiency, and economics ensures equity and motivation. The synergy among these three dimensions is essential for transforming integrative nutrition from a conceptual ideal into routine clinical practice, alleviating HCPs’ cognitive–emotional tension, strengthening patient adherence, and optimizing system-wide performance.

### 4.5 Limitations and future directions

This study has several limitations that should be acknowledged. First, although the multi-site design enhanced contextual diversity and strengthened the credibility of the findings, their generalizability remains constrained by the specific sociocultural and institutional setting. All participants were experienced HCPs recruited from five tertiary hospitals in Fujian Province, a region characterized by strong Southern Chinese cultural traits and a rich TCM heritage (46). Consequently, the findings are most applicable to Southern China and neighboring Southeast Asian regions with similar cultural contexts. Transferability to Northern China—where climate, dietary patterns, and TCM emphasis differ substantially (for example, heat-clearing versus dampness-dispelling therapeutic orientations)—requires further empirical verification.

Second, this study focused exclusively on the perspectives of HCPs, without including patients, family caregivers, or administrators. The absence of these viewpoints limits the multidimensional interpretation of the integrative nutrition ecosystem and the intersubjective dynamics within clinical practice.

Methodologically, the descriptive phenomenological approach entails an inherent interpretive co-construction between participants and researchers (18). While this study sought to manage rather than eliminate subjectivity, several measures were adopted to ensure analytical rigor and trustworthiness, including reflexive journaling, peer debriefing, dual coding of approximately 30% of transcripts (Cohen’s *κ* = 0.82), member checking, and maintenance of a transparent audit trail (47, 48). Third, purposive sampling may have preferentially attracted HCPs with stronger motivation, greater expertise, or more favorable attitudes toward integrative care, thereby introducing potential social desirability bias. Finally, as a cross-sectional study, it captures experiences at a single time point, limiting insight into the longitudinal evolution of cognitive–emotional tension and adaptive coping processes.

Future research should therefore employ longitudinal or mixed-methods designs to trace how HCPs’ psychological adjustment, professional identity, and patient outcomes evolve across different stages of integrative care. Such designs would enrich temporal understanding and enhance explanatory depth. Moreover, incorporating multi-stakeholder perspectives—including patients, families, and policy-makers—would enable a more holistic exploration of ICWNM’s implementation pathways, ultimately guiding evidence-based strategies for sustainable integration.

## 5 Conclusion

This study shows that healthcare professionals (HCPs) practicing Integrative Chinese–Western Nutritional Management (ICWNM) experience a persistent cognitive–emotional tension—a dynamic balance between professional fulfillment and pressure. While HCPs recognize the value of integrative care and gain satisfaction from improved patient outcomes, they simultaneously confront complex dual knowledge systems, insufficient structured training, and barriers to interdisciplinary collaboration. Capacity building remains overly dependent on individual initiative, creating a pervasive knowing–doing gap. Patient adherence is the critical bridge from intervention to outcomes, shaped by personal beliefs, cultural habits, and the surrounding system. At root, implementation challenges arise from structural deficits in policy, technology, and economic support.

Progress requires coordinated advances across three dimensions: policy to establish standardized pathways and regulatory frameworks; technology to enable knowledge translation and integrative decision-making; and economic mechanisms to provide equitable, sustainable incentives. The proposed HCPs (cognition–emotion)—patients (adherence)—system (support) framework offers a practical lens for designing sustainable, evidence-informed ICWNM models. Future work should adopt longitudinal, multi-center designs; incorporate patients, caregivers, and administrators; and leverage AI and mobile health (mHealth) tools to strengthen evidence generation and cross-cultural applicability—facilitating the transition of ICWNM from conceptual innovation to routine, sustainable practice.

## Data Availability

The original contributions presented in the study are included in the article/Supplementary material, further inquiries can be directed to the corresponding authors.
