# Supplementary material for: Exploring healthcare professionals’ views on integrative Chinese–Western medicine in the nutritional management of cancer patients: a qualitative study
Source: Front Nutr. 2026 Jun 10;13:1623146. doi: 10.3389/fnut.2026.1623146 (PMC13290459; doi:10.3389/fnut.2026.1623146)
Supplement: Supplementary file 1 [file Image_1.pdf]

Supplementary Material 3-Demographic characteristics of participating healthcare professionals

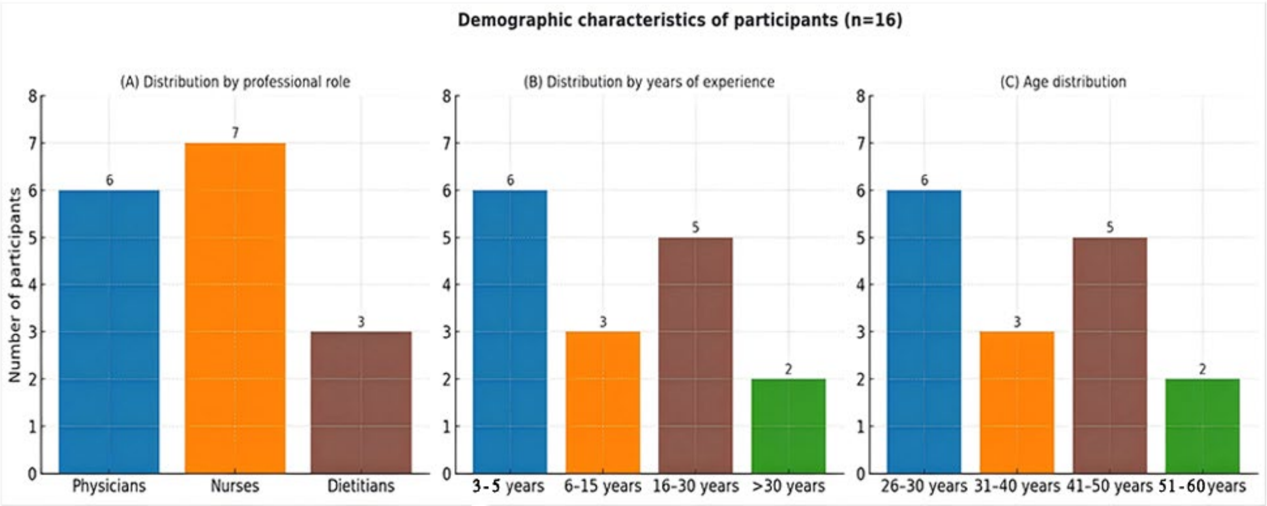

Supplementary Material 3. Demographic characteristics of participating healthcare professionals (n = 16).

Distribution of participants by (A) professional role (physicians, nurses, dietitians), (B) years of professional experience, and (C) age group.
