# Supplementary material for: Exploring healthcare professionals’ views on integrative Chinese–Western medicine in the nutritional management of cancer patients: a qualitative study
Source: Front Nutr. 2026 Jun 10;13:1623146. doi: 10.3389/fnut.2026.1623146 (PMC13290459; doi:10.3389/fnut.2026.1623146)
Supplement: Supplementary file 2 [file Table_1.docx]

Appendix 1. COREQ Checklist (Consolidated Criteria for Reporting Qualitative Research, 32 Items)

**Applicable Study:** This study adopted a phenomenological qualitative design to explore healthcare professionals’ (HCPs) experiences in the integrative Chinese–Western nutritional management (ICWNM) of cancer patients.

**Reference:** Tong A, Sainsbury P, Craig J. Consolidated criteria for reporting qualitative research (COREQ): a 32-item checklist for interviews and focus groups. Int J Qual Health Care. 2007;19(6):349–357.

# COREQ Checklist – Implementation in This Study

| No. | Item | Implementation in This Study |
| --- | --- | --- |
| 1 | Interviewer/facilitator | Interviews were conducted by two researchers The interviewers jointly participated in the data collection process. |
| 2 | Credentials | Both interviewers held postgraduate degrees and had formal academic training in integrative Chinese and Western medicine and qualitative research. |
| 3 | Occupation | At the time of the study, both interviewers were academically affiliated researchers engaged in clinical and research-related work. |
| 4 | Gender | Both interviewers were female. |
| 5 | Experience and training | Both interviewers had prior training in qualitative research and interviewing techniques. A pilot interview was conducted prior to formal data collection to refine the interview outline. |
| 6 | Relationship established | No prior personal relationship was established with participants before recruitment. |
| 7 | Participant knowledge of the interviewer | Participants were informed of the interviewers’ academic background, professional roles, and the purpose of the study prior to the interviews. |
| 8 | Interviewer characteristics | The interviewers maintained a neutral, non-judgmental stance and practiced reflexive awareness throughout the interviews. |
| 9 | Methodological orientation and Theory | This study adopted a phenomenological qualitative design. |
| 10 | Sampling | Participants were recruited using purposive sampling based on predefined eligibility criteria. |
| 11 | Method of approach | Participants were approached through professional networks and institutional contacts. |
| 12 | Sample size | Sixteen participants were included. |
| 13 | Non-participation | No participants withdrew after providing informed consent. |
| 14 | Setting of data collection | Interviews were conducted in a quiet and private setting convenient for participants. |
| 15 | Presence of non-participants | No non-participants were present during the interviews. |
| 16 | Description of sample | Participants were healthcare professionals with relevant clinical experience. |
| 17 | Interview guide | A semi-structured interview guide informed by literature review and research objectives was used. |
| 18 | Repeat interviews | Repeat interviews were not required. |
| 19 | Audio/visual recording | All interviews were audio-recorded with participants’ consent. |
| 20 | Field notes | Field notes were taken during and after interviews. |
| 21 | Duration | Interviews lasted approximately 30-60 minutes. |
| 22 | Data saturation | Data collection continued until data saturation was reached. |
| 23 | Transcripts returned | Interview transcripts were returned to participants for verification and confirmation. |
| 24 | Number of data coders | Two researchers independently participated in coding and analysis. |
| 25 | Description of the coding tree | Themes were generated through Colaizzi’s phenomenological analysis and refined through iterative team discussions. |
| 26 | Derivation of themes | Themes were derived inductively from the data. |
| 27 | Software | NVivo 12 software was used to support data management and coding. |
| 28 | Participant checking | Participant checking was conducted to enhance analytical credibility. |
| 29 | Quotations presented | Representative quotations were used to illustrate themes. |
| 30 | Data and findings consistent | Findings were grounded in participant narratives. |
| 31 | Clarity of major themes | Major themes were clearly presented and supported by data. |
| 32 | Clarity of minor themes | Minor themes and variations were described where relevant. |
