# Supplementary material for: Exploring healthcare professionals’ views on integrative Chinese–Western medicine in the nutritional management of cancer patients: a qualitative study
Source: Front Nutr. 2026 Jun 10;13:1623146. doi: 10.3389/fnut.2026.1623146 (PMC13290459; doi:10.3389/fnut.2026.1623146)
Supplement: Supplementary file 3 [file Table_2.docx]

## ****Appendix 2. Semi-Structured Interview Guide****

1. **Opening Statement**
   Thank you for agreeing to participate in this interview. We hope to learn about your experiences and perspectives regarding integrative Chinese and Western nutritional management in cancer care. There are no right or wrong answers—your personal insights are highly valuable. With your permission, this interview will be audio-recorded and all information will remain confidential and used only for academic research.

### ****2.Domain 1: Professional experiences and emotional perceptions****

**Q1.** How would you describe your role in providing integrative Chinese and Western nutritional care for cancer patients and what feelings or emotional experiences are associated with this work?

### ****3.Domain 2: Evolution of practice****

**Q2.** How has your approach to integrative nutritional management changed over time and what experiences or cases influenced these changes?

### ****3.Domain 3: Implementation challenges****

**Q3.** What challenges have you encountered when implementing integrative nutritional management and how have you or your team responded to these challenges?

### ****4.Domain 4: Professional training and peer sharing****

**Q4.** What kinds of training or professional support have been helpful in supporting your integrative nutritional care practice and what support or training do you feel is still lacking?

### ****5.Domain 5: Policy support and future expectations****

**Q5.** What policy or institutional support do you think is most needed to promote integrative nutritional management and what changes or improvements would you hope to see in the future?

**6.Closing Statement**

Thank you very much for your time and participation. Your insights are valuable for understanding and improving the integrative nutritional care model for cancer patients. Please feel free to add any additional comments before we conclude.
