# Supplementary material for: Exploring healthcare professionals’ views on integrative Chinese–Western medicine in the nutritional management of cancer patients: a qualitative study
Source: Front Nutr. 2026 Jun 10;13:1623146. doi: 10.3389/fnut.2026.1623146 (PMC13290459; doi:10.3389/fnut.2026.1623146)
Supplement: Supplementary file 4 [file Table_3.docx]

# Appendix 3 | Glossary of Key Terms

(This appendix provides standardized English translations and definitions of key concepts used throughout the study to ensure conceptual clarity and terminological consistency.)

| Term | Definition |
| --- | --- |
| Integrative Chinese–Western Nutritional Management (ICWNM) | A comprehensive model integrating Western nutrition science with Traditional Chinese Medicine (TCM) dietary therapy, emphasizing holistic regulation and individualized care. |
| Healthcare Professionals (HCPs) | Physicians, nurses, and dietitians engaged in providing integrative nutritional care for cancer patients. |
| Cognitive–Emotional Tension | A core concept describing the coexistence of professional fulfillment and stress among HCPs practicing ICWNM. |
| Patient Adherence | The extent to which patient behavior corresponds with agreed medical or nutritional advice. |
| Clinical Outcomes | Objective indicators reflecting improvement in clinical status, treatment tolerance, and quality of life. |
| Traditional Chinese Medicine Dietary Therapy (TCM Diet) | A dietary approach guided by syndrome differentiation and preventive principles rooted in TCM. |
| Pattern-Based Diet Formulation | Tailoring dietary interventions to syndrome differentiation for personalized nutrition. |
| Individualized Nourishment According to Body Constitution | Adjusting diet according to patients’ body constitution to maintain internal balance. |
| Preventive Principle of “Treating Disease Before Its Onset” | A preventive concept focusing on early intervention to maintain health. |
| Holistic Regulation | A systemic view emphasizing balance and coordination of body functions. |
| Qualitative Study | A research approach focusing on understanding experiences and meanings through textual data. |
| Phenomenological Qualitative Design | A qualitative approach aiming to describe participants’ lived experiences. |
| Semi-Structured Interviews | A flexible interview method combining predetermined questions with open-ended exploration. |
| Purposive Sampling | Selecting participants based on their relevance to the research question. |
| Colaizzi’s Seven-Step Phenomenological Analysis | A structured approach for analyzing qualitative data in phenomenological studies. |
| Data Saturation | The point at which no new information or themes emerge from the data. |
| Member Checking | A process in which participants verify the accuracy of data interpretation. |
| Dual Independent Coding | Two researchers independently code transcripts to ensure reliability. |
| Professional Fulfillment | A sense of accomplishment and meaning derived from professional practice. |
| Professional Burnout | A state of emotional exhaustion and diminished motivation. |
| Professional Resilience | The ability to recover from stress and sustain performance. |
| Interdisciplinary Collaboration | Cooperation among professionals from different fields to deliver integrated care. |
| Job Demands–Resources Model (JD-R Model) | A framework explaining how work demands and resources influence motivation and well-being. |
| Health Belief Model (HBM) | A behavioral model linking beliefs about illness to health behaviors. |
| Self-Determination Theory (SDT) | A motivation theory emphasizing autonomy, competence, and relatedness. |
| Conservation of Resources Theory (COR) | A framework explaining stress as the result of loss or threat of valued resources. |
| Continuing Professional Development (CPD) | Ongoing learning to maintain and enhance professional competence. |
| Comprehensive Supportive Ecosystem | A multidimensional system integrating policy, technical, and institutional support. |
| Standardized Clinical Pathway | Evidence-based structured steps for consistent patient care. |
| Mobile Health (mHealth) | The use of mobile technologies to support patient care and communication. |
| Clinical Decision Support System (CDSS) | A digital tool providing evidence-based recommendations for clinicians. |
| Professional Identity | The perception of belonging and commitment to one’s profession. |
| Workload | The amount and intensity of tasks expected in professional roles. |
| Cognitive Stress | Mental strain caused by complex or excessive work demands. |
| Capacity Building | Developing knowledge and skills required for effective practice. |
| Systematic Professional Training | Structured education to enhance clinical and theoretical competence. |
| Peer Knowledge Sharing | Mutual learning and experience exchange among peers. |
| Technological, Policy, and Economic Synergy | Coordination across technological, policy, and financial domains. |
| Sustainable Development | Ensuring the long-term viability of integrative healthcare practices. |
